# Fear Deficits in Hypomyelinated *Tppp* Knock-Out Mice

**DOI:** 10.1523/ENEURO.0170-20.2020

**Published:** 2020-09-23

**Authors:** Huy Nguyen, Lindsey M. Meservey, Nao Ishiko-Silveria, Mu Zhou, Ting-Ting Huang, Meng-meng Fu

**Affiliations:** 1 Department of Neurology, Stanford University School of Medicine, Stanford, CA 94305; 2 Department of Neurobiology, Stanford University School of Medicine, Stanford, CA 94305; 3 Department of Molecular and Cellular Physiology, Stanford University School of Medicine, Stanford, CA 94305; 4 Geriatric Research, Education, and Care Center, VA Palo Alto Health Care System, Palo Alto, CA 94304

**Keywords:** fear, microtubule, myelin, oligodendrocyte

## Abstract

Oligodendrocytes in the central nervous system (CNS) produce myelin sheaths that insulate axons to facilitate efficient electrical conduction. These myelin sheaths contain lamellar microtubules that enable vesicular transport into the inner sheath. Mechanistically, oligodendrocytes rely on Golgi outpost organelles and the associated protein tubulin polymerization promoting protein (TPPP) to nucleate or form new microtubules outside of the cell body. Consequently, elongation of lamellar microtubules is defective in *Tppp* knock-out (KO) mice, which have thinner and shorter myelin sheaths. We now explore the behavioral phenotypes of *Tppp* KO mice using a number of different assays. In open-field assays, *Tppp* KO mice display similar activity levels and movement patterns as wild-type mice, indicating that they do not display anxiety behavior. However, *Tppp* KO mice lack fear responses by two types of assays, traditional fear-conditioning assays and looming fear assays, which test for innate fear responses. Deficits in fear conditioning, which is a memory-dependent task, as well as in spatial memory tests, support possible short-term memory defects in *Tppp* KO mice. Together, our experiments indicate a connection between CNS myelination and behavioral deficits.

## Significance Statement

Oligodendrocytes are cells in the brain that make myelin sheaths, which wrap concentrically around axons to provide insulation that facilitates electrical conduction. Fear responses have historically been attributed to neuronal activity. However, emerging literature indicates that mouse models with defective myelination display long-term fear deficits. Here, we look at a specific mouse model that lacks an oligodendrocyte-specific protein that is important for building the cellular structure of microtubules, which allow for transport to and along the myelin sheath. These mice display deficits in both memory-dependent fear as well as innate fear responses. Thus, our work indicates that myelin structure is important for fear response.

## Introduction

Oligodendrocytes insulate neuronal axons in a lipid-rich covering called myelin that facilitates fast axonal conduction velocities. Defects in myelination contribute to diseases, including multiple sclerosis and leukodystrophies. In addition, faulty myelination has been previously implicated in psychiatric diseases, including schizophrenia, depression, bipolar disorder, and obsessive-compulsive disorder (Fields, 2008).

Both neuroimaging and genetics data connect white matter changes with schizophrenia, a mental disorder that can manifest in hallucinations and delusions. Magnetic resonance imaging (MRI) studies have indicated a disruption in white matter structure in schizophrenic patients (Foong et al., 2000; Kubicki et al., 2005; Fields, 2008). In addition, polymorphisms associated with schizophrenia are found in genes encoding oligodendrocyte transcription factor 2 (OLIG2; Georgieva et al., 2006), which is crucial for oligodendrocyte development, and 2’,3’-cyclic nucleotide 3’-phosphodiesterase (CNP), a structural myelin protein (Hagemeyer et al., 2012).

Many studies of human schizophrenic brains have observed changes to myelin or oligodendrocytes. Electron microscopy (EM) of prefrontal cortex (PFC) of schizophrenic brains indicates damage to myelin sheath lamellae and changes in oligodendrocyte organelle content (Uranova et al., 2001). The frontal gyrus of schizophrenic patients contains ∼20% fewer oligodendrocytes than those of control patients (Hof et al., 2002). Multiple microarray studies of both prefrontal and temporal cortex of schizophrenic brains found significant downregulation of genes involved in myelination and oligodendrocyte development (Hakak et al., 2001; Tkachev et al., 2003; Aston et al., 2004). These studies raise the intriguing hypothesis that defects in oligodendrocyte development and myelination may underlie symptoms in schizophrenia.

In mice, several recent studies suggest a link between myelination defects and behavioral phenotypes. Mice exposed to cuprizone, a demyelinating agent, exhibit deterioration of oligodendrocytes in the PFC and schizophrenia-like symptoms (Gregg et al., 2009). Mass spectrometry experiments elucidated transient changes in myelin proteins ∼24 h following contextual fear conditioning (Houyoux et al., 2017), indicating that myelin remodeling may be involved in short-term fear response.

Evidence from genetic mouse models suggest that enhanced myelination heightens fear-conditioned learning while impaired myelination impedes fear-conditioned learning. For example, activation of ERK1/2 (extracellular signal-regulated kinase) in oligodendrocytes increased myelination throughout the CNS and increased contextual response after fear conditioning (Jeffries et al., 2016). On the other hand, mice in which the transcription factor MYRF (myelin regulatory factor) was knocked out in oligodendrocyte precursor cells (OPCs) display long-term defects in freezing (Pan et al., 2020). In addition, loss of the developmental regulator *Cdk5* (cyclin-dependent kinase 5) in mature oligodendrocytes reduces myelination and fear-based learning (Luo et al., 2018). However, as ERK, CDK5, and MYRF broadly regulate cell function, it is unclear whether structural myelin changes underlie these changes.

Our study mechanistically illuminates the relationship between myelination and fear-based behaviors using a mouse in which hypomyelination has been attributed to structural defects in microtubules. Oligodendrocytes contain two classes of microtubules, radial microtubules that are proximal to the cell body and extend toward axons, and lamellar microtubules that are distal and spiral around the myelin sheath (Weigel et al., 2020). Oligodendrocytes rely on the formation or nucleation of microtubules outside the cell body off of Golgi outpost organelles (Valenzuela et al., 2020). This nucleation function is performed by tubulin polymerization promoting protein (TPPP; Fu et al., 2019). *Tppp* mRNA is highly expressed by oligodendrocytes, but extremely low in other brain cells (Zhang et al., 2014; Schaum et al., 2018) and in other non-brain tissues (Schaum et al., 2018).

A previous study found that both cultured and *in vivo* oligodendrocytes from homozygous *Tppp* knock-out (KO) mice have shorter lamellar microtubules and consequently shorter and thinner myelin sheaths (Fu et al., 2019). Cultured *Tppp* KO oligodendrocytes have additional aberrant features, including more proximal branches, mixed microtubule polarity and accumulation of myelin basic protein (*Mbp*) mRNA, an abundantly transported cargo (Carson et al., 1997; Herbert et al., 2017). Furthermore, immunostaining against MBP protein shows that *Tppp* KO brains have decreased myelination in spinal cord, caudate, cortex, and hippocampus, with upper cortical layers and CA1 and CA3 fiber tracts that are strikingly devoid of myelination. No gross differences were observed in neurofilament staining, indicating that axonal tracts and neuronal morphology is largely intact in *Tppp* KO mice (Fu et al., 2019).

Our current research explores fear-based behaviors in these myelin-deficient mice. In open-field assays and light-dark preference assays, *Tppp* KO mice display similar activity levels as wild-type mice, indicating lack of anxiety behavior. In contrast, *Tppp* KO mice are profoundly deficient in fear responses. We use two types of assays, traditional fear conditioning and looming fear, to evaluate memory-dependent fear responses and innate fear responses, respectively. Results from both fear conditioning and spatial memory tests support short-term memory deficits in *Tppp* KO mice. Altogether, our study further strengthens the connection between myelination and fear-based learning and adds an additional connection to innate fear.

## Materials and Methods

### Animals

All animal procedures were performed in accordance with the Stanford University animal care committee’s regulations. *Tppp* KO mice were recovered from sperm from the Knockout Mouse Project (KOMP; stock number VG12652) and bred in the C57BL/6J strain. Animals were bred from heterozygous parents and age-matched littermates were selected for behavioral assays. Only three-month-old male mice were used for behavioral experiments. Mice that underwent fear conditioning were subject either to this assay alone or after all other behavioral tests were completed. Mice were tested blinded and the genotypes of mice were only revealed to the experimenter after the end of the experiment. All data represent one observation per animal per assay as additional consecutive trials would reflect habituation rather than an independent biological replicate.

### Open field activity assay

An individual mouse was placed in a new cage inside the SmartCage platform (AfaSci) for 1 h. The animal’s position and locomotion were determined by breakages in the infrared (IR) beams provided by the SmartCage platform. Measurements of activity counts (i.e., breaks in the IR beams in the *x*-, *y*-, and/or *z*-directions) were automatically quantified using the accompanying CageScore software (Khroyan et al., 2012).

### Light-dark box assay

To assess anxiety-related behavior, a dark red box made of Plexiglas with a small entrance was placed inside the mouse cage and extra overhead lighting was provided outside this box, separating the cage into a light compartment and a dark compartment. The amount of time the animal spent in the light or dark compartment was quantified by CageScore (Khroyan et al., 2012) over a period of 1 h.

### Trace fear conditioning

Individual mice were placed inside the fear conditioning chamber (Coulbourn Instruments) which was cleaned with 10% ethanol as background odor. A ventilation fan provided ∼55 dB of background noise. Mice were preexposed to the chamber for 5 min 24 h before training. On training day (day 1, 24 h later), after a 4-min period of exploration to establish baseline behavior, a 85-dB 2-kHz tone was played for 20 s (cue period), followed by an 18-s trace period and a 2-s foot shock of 0.75 mA. The process was repeated four times and separated by 1-min intertrial intervals (ITIs).

On context recall day (day 2, 24 h later), mice were placed back in the training chamber for 4 min. On altered context testing day (day 3, 24 h later), mice were placed in an altered conditioning chamber (side walls decorated with different patterns, smooth solid plastic floor, 1% vanilla background odor); the same protocol used on training day minus the foot shock was applied. The behavior of the mice was recorded and analyzed with FreezeView software (Coulbourn Instruments). On training day, the freezing percentages during each exploration, cue, trace, and ITI period were summarized as an indication of fear memory acquisition. On context recall day, the freezing percentage was compared with that of the last ITI of training day. On altered context testing day, the averaged freezing percentages of cue, trace, and ITI periods were reported.

Fear-conditioned mice were not subsequently used for any other assays.

### Y-maze

To assess working spatial memory, an acrylic Y-shaped maze consisting of three symmetrical arms (20 × 8 × 16 cm) at 120° angles was used as the testing arena. The end of each arm of the maze was decorated with a different visual cue. Individual mice were placed at the center of the maze and allowed to freely explore the three arms for 5 min. Over the course of the testing time, wild-type mice are expected to display a propensity to explore a new arm of the maze instead of re-entering a previously visited arm. Arm entry was scored when the animal reached the end of the arm of the maze. The total number of entries and the number of unique triad combination of consecutive arm entries (ABC, ACB, BAC, BCA, CAB, CBA) were manually scored. The percentage of alteration (spontaneous alteration) was calculated as the number of unique entries/(total number of entries – 2).

### Looming fear assay

Looming behavior was measured as previously described (Salay et al., 2018). Briefly, a mouse was placed in a 50 × 25 × 40 cm glass chamber with a 12-cm shelf at one end of the chamber to provide a shelter for mice to hide. At the top of the cage, a 24-inch LCD monitor facing onto the cage displayed stimuli. A video camera was placed outside the cage to record the mouse’s movements. Mice were introduced to the cage and allowed to habituate for ∼10 min. Then a looming stimulus (expanding black disk on a white background, 15 expansions over 24 s) was projected on the LCD monitor.

Video recordings were analyzed by bins of 1 s. The percentage of time spent hiding, freezing, running, or ambulating was calculated. Hiding was classified as the mouse’s entire body being underneath the shelf. Freezing was classified as episodes of >3 s of no movement except for respiration. Running was classified as greater than two times the average ambulatory speed. All other motile behaviors were classified as ambulation.

The number of rearing events and head tilts were individually scored (i.e., they are not part of the percentage calculations above). Head tilts were defined as raising the head >20° (relative to the main body axis with vertex at the nape of the neck).

### Pupillary light reflex (PLR)

Mice were dark-adapted for 1 h before measurement. Mice were manually restrained and placed in front of a video camera. After 5 s, blue light was placed in front of the left pupil for 35 s, after which the pupils were allowed to recover. Pupil size was analyzed using ImageJ and normalized to total eye size.

### Novel female interest assay

As previously described (Jones et al., 2019), a cardboard box was used as the testing arena for female location memory test. On day 0, 24 h before female exposure, individual male mice were allowed to freely explore the testing arena for 10 min. On day 1 (experience day), a novel female mouse (unfamiliar to the male mice) was randomly placed inside one of the four slotted metallic enclosures placed at the corners of the testing arena. Male mice were placed at the center of the arena facing the visual cues on the wall and subjected to 10 min of experience trial. The amount of time the male mice spent interacting with the female inside the enclosure was scored as female interest.

### Statistical analysis

All data are presented as mean ± SEM (standard error of the mean) and were analyzed with Prism 8 (GraphPad Software). Data were evaluated for normality using Kolmogorov–Smirnov test. For data with two conditions, parametric data were analyzed using unpaired *t* test; non-parametric data were analyzed using Mann–Whitney (Wilcoxon rank-sum test). All other data were analyzed using two-way ANOVA (analysis of variance) with *post hoc* Sidak’s multiple comparison test to compare wild-type to *Tppp* KO mice values; *p* < 0.05 was considered significant, and the following notations were used to indicate significance: *p < 0.05, **p < 0.01, ***p < 0.001, ****p < 0.0001.

## Results

### Open field and light-dark box tests

**Figure 1.**
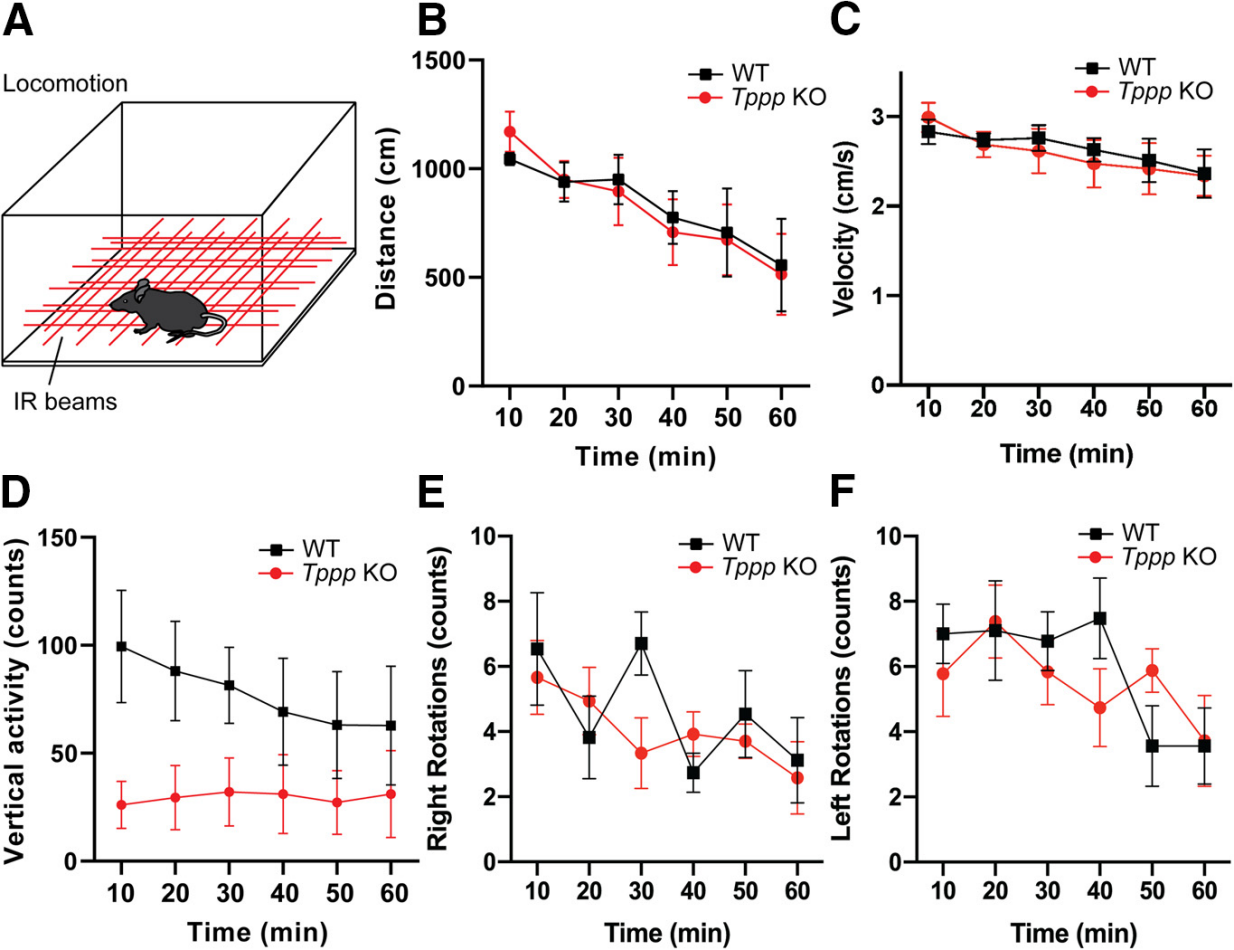
*Tppp* KO mice display normal open field activity. ***A***, Open field assay experimental design. Wild-type and *Tppp* KO mice were allowed to move freely through an arena where IR beams measured their movement; *n* = 5 mice per genotype for all data in this figure. Detailed statistical results can be found in Extended Data Figure 1-1; power analyses can be found in Extended Data Figure 1-2. ***B***, Wild-type and *Tppp* KO mice did not show any differences in distance traveled during the 60-min locomotion test. ***C***, Wild-type and *Tppp* KO mice did not show any differences in velocity during the 60-min locomotion test. ***D***, Wild-type and *Tppp* KO mice did not show any significant differences in number of vertical activity events when evaluated by two-way ANOVA (*p* = 0.170, time × genotype interaction). Evaluating wild-type versus *Tppp* KO means at each time point using independent *t* tests yielded *p* = 0.016 for the first time point (wild type: 99.4 counts, *Tppp* KO: 26.0 counts) but *p* > 0.05 for all other timepoints, suggesting that wild-type mice show significantly more vertical activity during the first 10 min of the 60-min locomotion test. Graph represent significance based on two-way ANOVA with *post hoc* Sidak’s multiple comparisons test. ***E***, Wild-type and *Tppp* KO mice did not show any significant differences in number of right rotations during the 60-min locomotion test. At the 30-min time point, *p* = 0.209 (*p* = 0.0504 by independent *t* test of means at that time point). ***F***, Wild-type and *Tppp* KO mice did not show any differences in number of left rotations during the 60-min locomotion test. At the 40-min time point, *p* = 0.4741 (*p* = 0.149 by independent *t* test of means at that time point) and at the 50-min time point, *p* = 0.6586 (*p* = 0.148 by independent *t* test of means at that time point). At all other time points, *p* > 0.45. Error bars represent SEMs.

We first examined the baseline activity level of *Tppp* KO mice using a 60-min open field test in which intersecting IR beams were used to measure lateral and vertical translocation (Gould et al., 2009; Fig. 1*A*). There was no significant difference in total horizontal distance or velocity traversed by *Tppp* KO mice compared with their wild-type counterparts (Fig. 1*B*,*C*; Extended Data Figs. 1-1, 1-2), indicating similar levels of ambulation and activity. *Tppp* KO mice did not show significant differences in vertical activity (Fig. 1*D*). No differences in the number of right or left rotations were observed between *Tppp* KO mice and wild-type mice (Fig. 1*E*,*F*; Extended Data Figs. 1-1, 1-2), indicating no evidence of aberrant twirling or spinning behaviors.

10.1523/ENEURO.0170-20.2020.f1-1 Extended Data Figure 1-1 Detailed results of statistical analysis in Figure 1. Download Figure 1-1, XLSX file.

**Figure 2.**
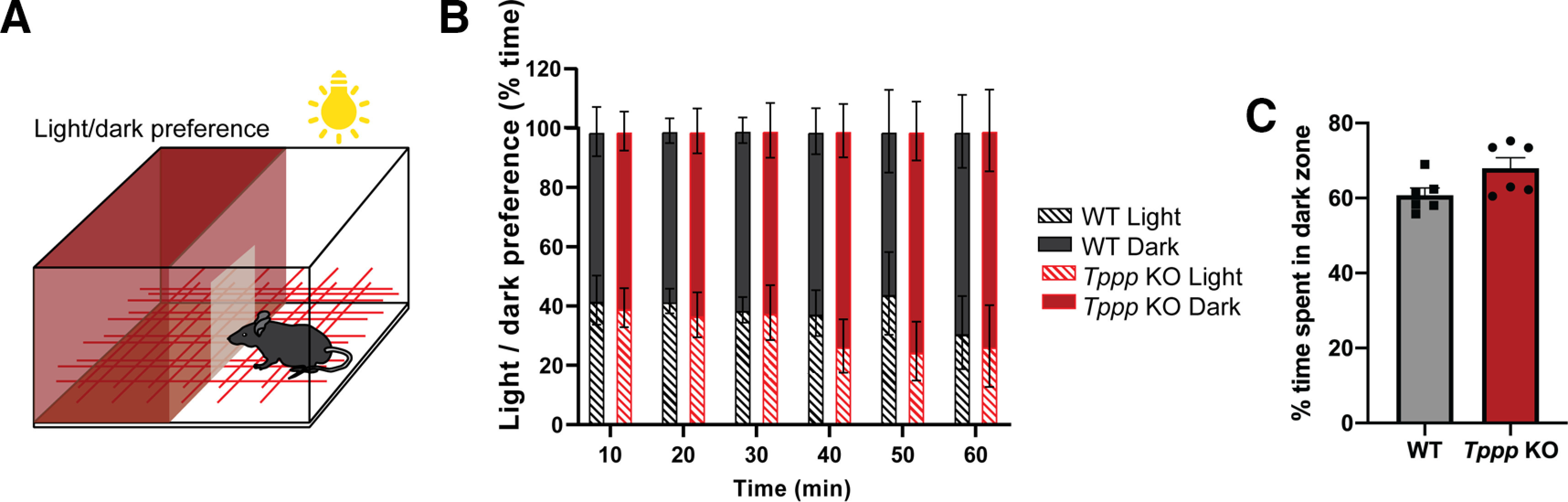
*Tppp* KO mice display normal light-dark preferences. ***A***, Light-dark box assay experimental design. Wild-type and *Tppp* KO mice were allowed to move freely between light and dark sides of a cage to examine anxiety; *n* = 5 mice per genotype for all data in this figure. Detailed statistical results can be found in Extended Data Figure 2-1; power analyses can be found in Extended Data Figures 1-2, 2-2. ***B***, Wild-type and *Tppp* KO mice did not show any difference in percentage of time spent in light or dark zones when analyzed in 10-min bins. ***C***, Wild-type and *Tppp* KO mice did not show any difference in total percentage of time spent in the dark zone. Error bars represent SEMs.

10.1523/ENEURO.0170-20.2020.f1-2 Extended Data Figure 1-2 Power, two-way ANOVAs. Download Figure 1-2, XLSX file.

We also examined anxiety-like behavior in *Tppp* KO mice using a light-dark box test (Fig. 2*A*), in which anxious mice are expected to spend more time in darker areas than in lit areas (Crawley, 1985). There was no significant difference in the percentage of time spent in the dark zone when analyzed by 10-min bins or by total time between *Tppp* KO and wild-type mice (Fig. 2*B*,*C*; Extended Data Figs. 2-1, 2-2), suggesting normal levels of unconditioned or inherent anxiety in *Tppp* KO mice.

10.1523/ENEURO.0170-20.2020.f2-1 Extended Data Figure 2-1 Detailed results of statistical analysis in Figure 2. Download Figure 2-1, XLSX file.

10.1523/ENEURO.0170-20.2020.f2-2 Extended Data Figure 2-2 Power, *t* tests. Download Figure 2-2, XLSX file.

### Fear conditioning assay

**Figure 3.**
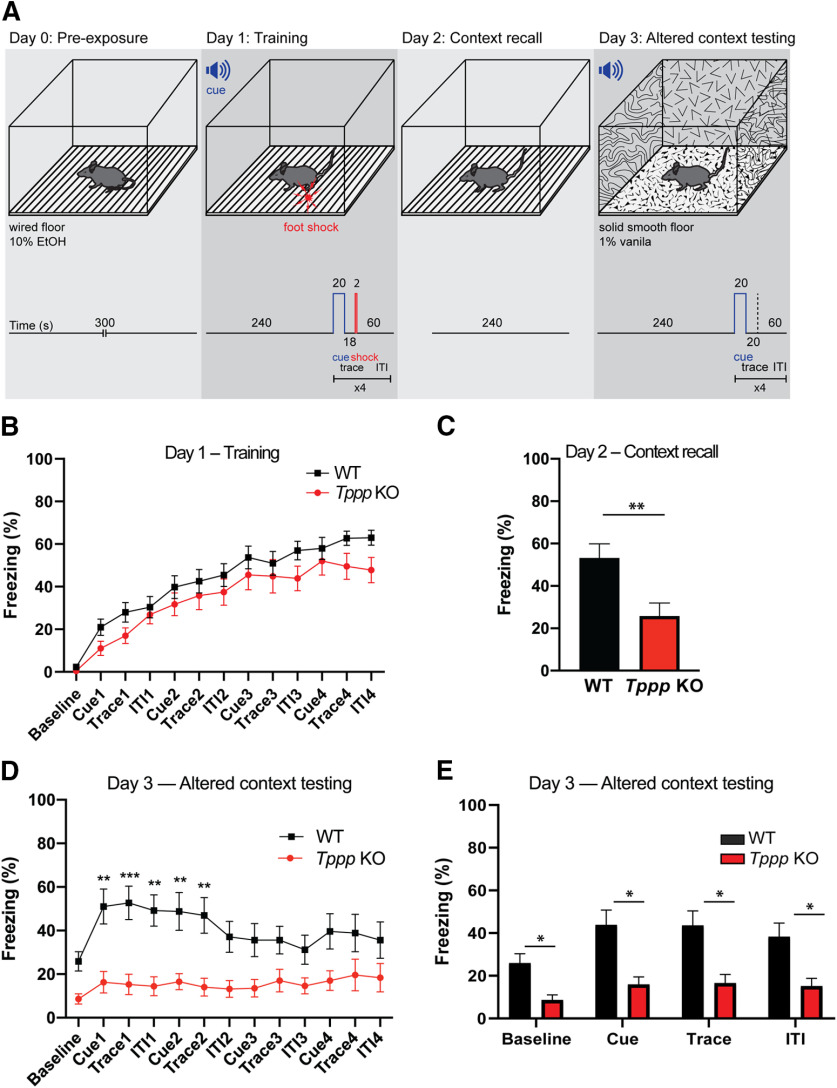
*Tppp* KO mice display defective fear conditioning. ***A***, Fear conditioning assay experimental design. On day 0, mice were acclimated to a wired-floor cage with a background scent of 10% ethanol. On day 1, the mice were again acclimated to the cage and then a cue sound was played for 20 s followed by a foot shock and a 60-s ITI. The cue, trace, shock, and ITI were repeated for a total of four times. On day 2, mice were placed in the same wired floor cage with 10% ethanol scent. On day 3, mice were acclimated to a cage with solid smooth flooring and 1% vanilla background scent and then given the cue sound, without the shock; *n* = 11 mice per genotype for all data in this figure. Detailed statistical results can be found in Extended Data Figure 3-1; power analyses can be found in Extended Data Figures 1-2, 2-2. ***B***, On day 1 (training), *Tppp* KO mice had similar freezing responses as wild-type mice, with no significant differences in percentage time spent freezing when evaluated by two-way ANOVA with *post hoc* Sidak’s multiple comparisons test. Graphs represent significance based on two-way ANOVA with *post hoc* Sidak’s multiple comparisons test. Evaluating wild-type versus *Tppp* KO means as independent *t* tests (i.e., at different time points/periods) gives *p* values of 0.371 (wild type: 2.306%, *Tppp* KO: 0.472%) at baseline and of 0.042 (wild type: 62.94%, *Tppp* KO: 47.79%) during ITI4 (*p* = 0.042). All other *p* values generated by independent *t* tests were >0.05. ***C***, On day 2 (context recall), *Tppp* KO mice displayed less freezing overall compared with wild-type mice. *p* = 0.007 by Welch’s *t* test. ***D***, On day 3 (context recall), *Tppp* KO mice displayed significantly less freezing than wild-type mice at all timepoints during the first two rounds of training. Graphs represent significance based on two-way ANOVA with *post hoc* Sidak’s multiple comparisons test. Evaluating wild-type versus *Tppp* KO means as independent *t* tests (i.e., at different time points/periods) reveals significant *p* values for all other timepoints except trace 3: at baseline, *p* = 0.0014 (wild type: 24.74%, *Tppp* KO: 8.650%); for cue 3, *p* = 0.0213 (wild type: 35.65%, *Tppp* KO: 13.55%); for trace 3, *p* = 0.0634 (wild type: 35.64%, *Tppp* KO: 17.06%); for ITI 3, *p* = 0.0473 (wild type: 31.21%, *Tppp* KO: 14.60%); for cue 4, *p* = 0.0269 (wild type: 39.66%, *Tppp* KO: 17.03%); for trace 4, *p* = 0.0294 (wild type: 38.85%, *Tppp* KO: 19.64%); for ITI 4, *p* = 0.0320 (wild type: 35.63%, *Tppp* KO: 18.36%). ***E***, On day 3, *Tppp* KO mice displayed significantly less freezing during baseline, cue, trace, and ITI periods than wild-type mice; *p* values calculated by two-way ANOVA. Error bars represent SEMs. *p < 0.05, **p < 0.01, ***p < 0.001.

Having established normal anxiety and baseline activity levels, we examined differences in fear conditioning learning between *Tppp* KO and wild-type mice using a context-dependent and cue-dependent trace conditioning assay (Fig. 3*A*). On day 0 of this assay, mice were acclimated to a new environment. On day 1, mice were returned to this same environment, and their baseline activity was assessed. Our data indicate that *Tppp* KO and wild-type animals display similar levels of low or no baseline freezing (Fig. 3*B*), which is consistent with open field assays in which *Tppp* KO mice have similar levels of locomotion as wild-type mice (Fig. 1*B*). Animals are then exposed to an auditory cue followed by a noxious stimulus (foot shock). Although at 85 dB this tone is not loud enough to stimulate an acoustic startle response (Pantoni et al., 2020), both wild-type and *Tppp* KO mice increase the percentage of time spent freezing. This indicates that *Tppp* KO mice can perceive the tone (i.e., are not deaf) and furthermore that they freeze acutely in response to the tone. As four successive cycles of cue and shock proceed, both wild-type and *Tppp* KO mice respond by increasing the percentage of time spent freezing (Fig. 3*B*). Interestingly, looking individually at periods during the fourth and last cycle, *Tppp* KO mice surprisingly display slightly lower percentages of time spent freezing during the period following the final auditory cue (ITI4, by *t* test), although these differences are not significant by two-way ANOVA (Fig. 3*B*; Extended Data Fig. 3-1).

10.1523/ENEURO.0170-20.2020.f3-1 Extended Data Figure 3-1 Detailed results of statistical analysis in Figure 3. Download Figure 3-1, XLSX file.

Movie 1. Looming fear in a wild-type mouse. Video recording of looming fear assay performed on a wild-type mouse. 10.1523/ENEURO.0170-20.2020.video.1

Movie 2. Looming fear in a *Tppp* KO mouse. Video recording of looming fear assay performed on a *Tppp* KO mouse. 10.1523/ENEURO.0170-20.2020.video.2

On the following days, mice are assessed for context-based recall and cue-based recall. On day 2, mice are returned to the same environment, but with no auditory cue or foot shock (Fig. 3*A*) and context recall is measured by their fear response in terms of time spent freezing. Wild-type animals displayed similar levels of freezing (Fig. 3*C*) as day 1 during the last two cycles of training (Fig. 3*B*). Remarkably, on day 2, *Tppp* KO mice spent about half as much time frozen (25.8%) compared with wild-type mice (53.2%); this difference (27.4% less) had a 95% margin of error of 9.1% (Fig. 3*C*). Interestingly, *Tppp* KO mice spent a similar percentage of time frozen as they did during the first cycle of shocks on day 1 (Fig. 3*B*). This indicates a significant degradation of the contextual memory in *Tppp* KO mice when they were returned to the conditioning chamber.

On day 3, to evaluate cue-based recall, mice were introduced to a new environment but the auditory cue is played again for four cycles with the same timing as day 1 (Fig. 3*A*). These results were analyzed for the individual periods of each cycle (Fig. 3*D*) and as binned results for each period (Fig. 3*E*). Baseline freezing levels were lower in *Tppp* KO mice before the auditory cue (Fig. 3*E*), consistent with day 2 context recall results (Fig. 3*C*). During the cue recall, wild-type mice froze ∼40–50% of the time during the cue sound, during the trace period immediately following the cue, and during the ITI before the next cycle. In contrast, *Tppp* KO mice spend significantly less time freezing, only ∼10–15% (Fig. 3*E*). These differences are most profound during the first two cycles (Fig. 3*D*). Thus, the lack of a significant freezing response in *Tppp* KO mice after the auditory tone suggests a severe impairment in their cued memory recall.

Strikingly, *Tppp* KO mice froze significantly less than wild-type mice during all stages of this assay, initial fear conditioning on day 1 (Fig. 3*B*), context-based recall on day 2 (Fig. 3*C*), and cue-based recall on day 3 (Fig. 3*D*,*E*). Together, these results are consistent with a robust defect in fear conditioning.

**Figure 4.**
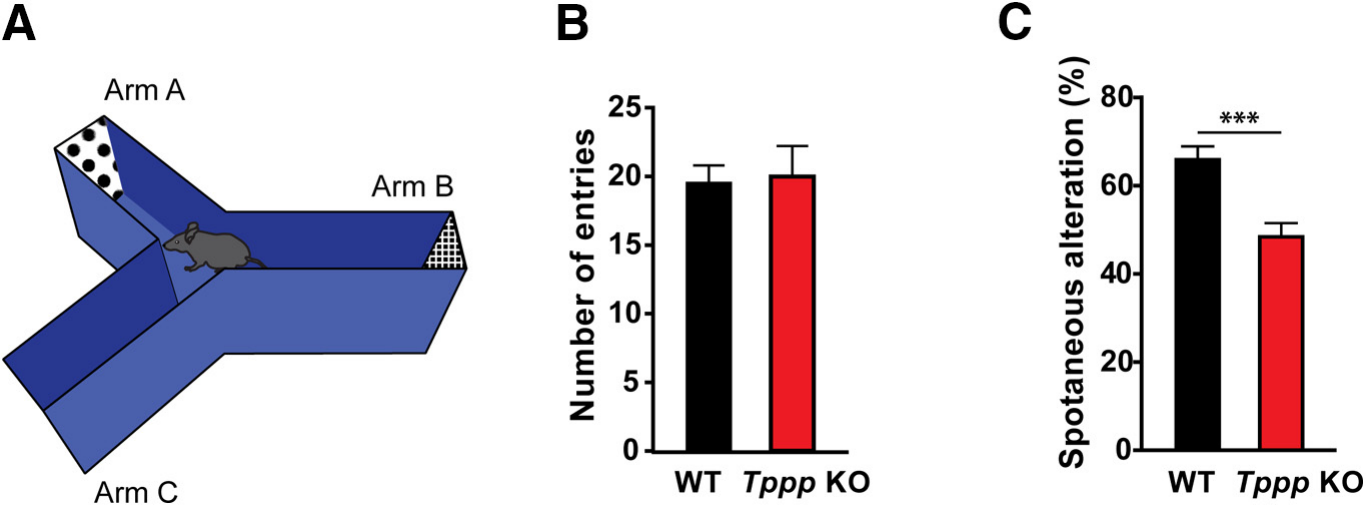
*Tppp* KO mice have spatial memory defects. ***A***, Y-maze assay experimental design. Wild-type and *Tppp* KO mice were placed in the center of a Y-shaped apparatus and allowed to travel to one of its arms, each with a different visual cue at the end; *n* = 8 mice per genotype for all data in this figure. Detailed statistical results can be found in Extended Data Figure 4-1; power analyses can be found in Extended Data Figure 2-2. ***B***, *Tppp* KO and wild-type mice did not show a significant difference in number of total entries. ***C***, *Tppp* KO mice had lower percentage of spontaneous entry alteration than wild-type counterparts; *p* = 0.0014 by Welch’s *t* test. Error bars represent SEMs. ***p < 0.001.

We next asked whether the attenuated contextual fear memory retention in *Tppp* KO mice could be a result of spatial memory deficit using the Y-maze test (Fig. 4*A*). We observed that wild-type mice and *Tppp* KO mice had no difference in total number of entries (Fig. 4*B*; Extended Data Fig. 4-1), perhaps consistent with similar distances traversed in the open field assay (Fig. 1*B*). However, *Tppp* KO mice had significantly lower percentage of unique entry combinations (i.e., spontaneous alternating behavior) than their wild-type counterparts (Fig. 4*C*), suggesting deficits in working spatial memory.

10.1523/ENEURO.0170-20.2020.f4-1 Extended Data Figure 4-1 Detailed results of statistical analysis in Figure 4. Download Figure 4-1, XLSX file.

### Looming fear assay

**Figure 5.**
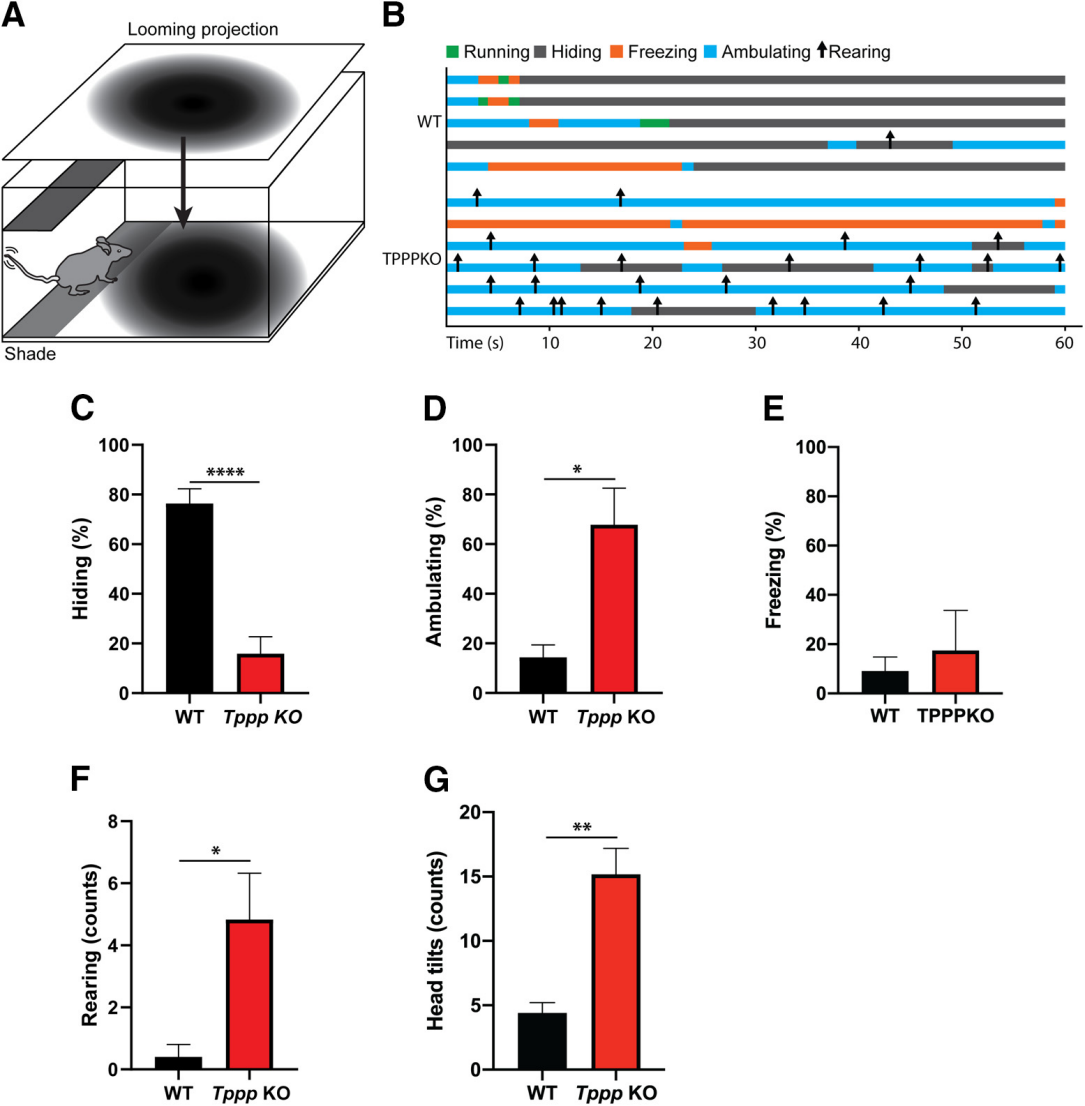
*Tppp* KO mice lack looming fear response. ***A***, Looming fear assay experimental design. Mice were placed in an arena with a shelf on one side to hide under. The ceiling of the arena contained an LED screen that projected a looming stimulus, an expanding black circle on a white background, onto the cage. Detailed statistical results can be found in Extended Data Figure 5-1; power analyses can be found in Extended Data Figure 2-2. ***B***, Ethogram of responses to looming stimulus, classified as either running, hiding, freezing, or ambulating. Arrows represent rearing events. Running events were only observed in wild-type mice; *n* = 5 wild-type mice, *n* = 6 *Tppp* KO mice for all data in this figure. ***C–E***, Percentage of time spent hiding, ambulating or freezing in response to looming stimulus. Compared with wild-type mice, *Tppp* KO mice spent significantly less time hiding (*p* = 0.0001), significantly more time ambulating (*p* = 0.011), and no significant difference in time freezing (*p* = 0.26). ***F***, *Tppp* KO mice displayed significantly more rearing events than wild-type mice (*p* = 0.030). ***G***, *Tppp* KO mice displayed significantly more head tilts than wild-type mice. Error bars represent SEMs. *p < 0.05, **p < 0.01, ****p < 0.0001.

We next sought to understand whether *Tppp* KO mice also have decreased innate fear response to natural threats using the looming fear assay. This assay tests innate visual fear response using a looming screen that projects a dark, rapidly expanding round object on a white background that simulates a swooping predator (Fig. 5*A*; Salay et al., 2018). We binned mouse behavior during the looming stimulus into freezing, hiding, running, and ambulating (Fig. 5*B–E*; Extended Data Fig. 5-1; Movies 1, 2). While wild-type mice spent the majority of time hiding (76.3%), *Tppp* KO mice spent significantly less time hiding (15.8%); this difference of 60.5% had a 95% margin of error of 9.1% (Fig. 5*C*). In contrast, *Tppp* KO mice spent the majority of time ambulating (67.8%), which was significantly higher than in wild-type mice (14.3%); this difference of 53.4% had a 95% margin of error of 15.6% (Fig. 5*D*). No statistically significant difference was observed for the percentage of time spent freezing, although we calculated a low power for this comparison, possibly indicating a false negative result (Fig. 5*E*; Extended Data Fig. 2-2).

10.1523/ENEURO.0170-20.2020.f5-1 Extended Data Figure 5-1 Detailed results of statistical analysis in Figure 5. Download Figure 5-1, XLSX file.

In addition, we observed incidents of rearing and head tilting in *Tppp* KO mice and quantified these in terms of number of events. Surprisingly, *Tppp* KO mice also had significantly more rearing events when compared with wild-type mice, in which only one rearing event was observed across all animals (Fig. 5*F*). In addition, *Tppp* KO mice also displayed more head tilting events (15.2) compared with wild-type mice (4.4); this difference (10.8 more) had a 95% margin of error of 2.2 (Fig. 5*G*). Both rearing and head tilting behavior appear to be attempts to look at the looming stimulus. Together, results from this assay suggest a robust deficit in innate fear response in *Tppp* KO mice.

**Figure 6.**
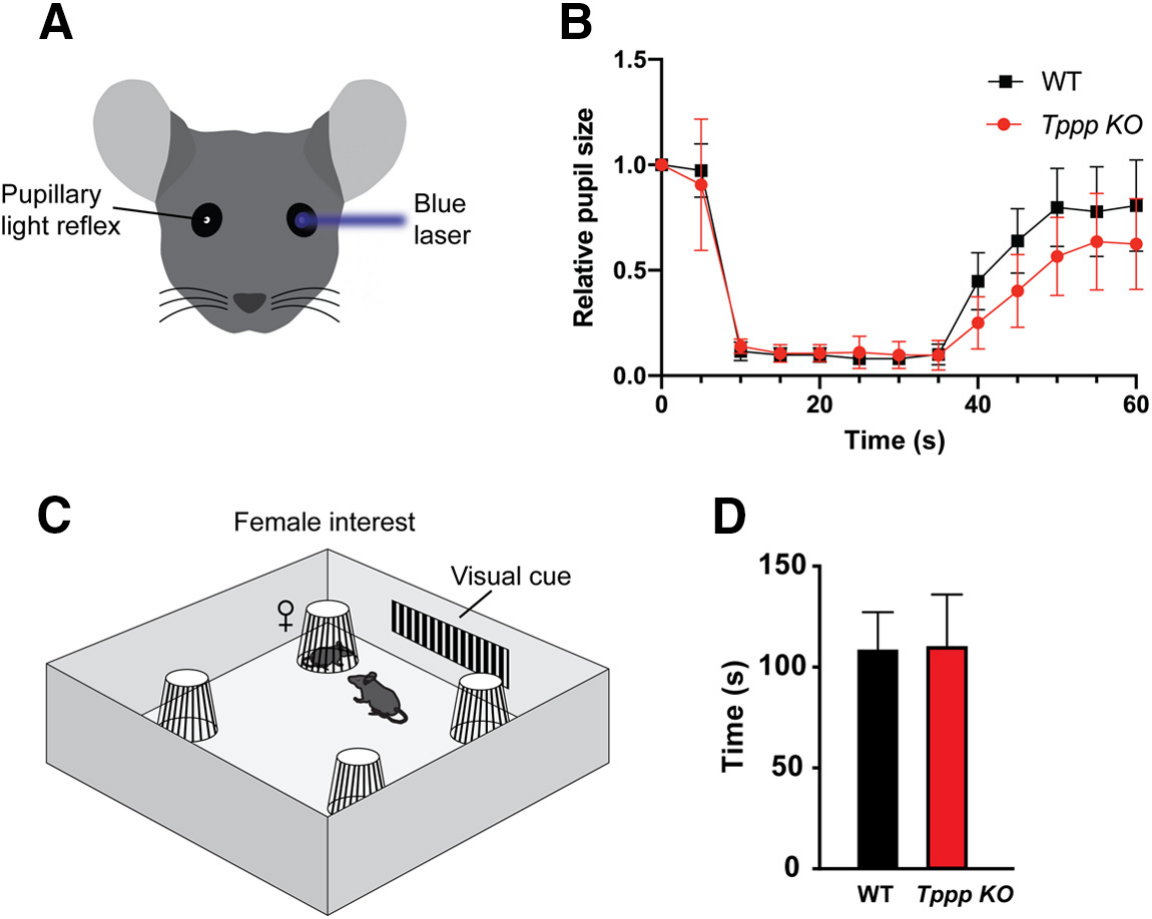
*Tppp* KO mice have normal pupillary dilation. ***A***, Pupillary dilation assay experimental design. A blue laser was beamed into the left pupil and the light reflex of the right pupil was recorded. Detailed statistical results can be found in Extended Data Figure 6-1; power analyses can be found in Extended Data Figures 1-2, 2-2. ***B***, Both wild-type and *Tppp* KO mice display pupil dilation reflexes. Graphs represent significance based on two-way ANOVA with *post hoc* Sidak’s multiple comparisons test, which shows no significant difference in relative pupil size between *Tppp* KO and wild-type mice. Evaluating wild-type versus *Tppp* KO means as independent *t* tests (i.e., at different time points/periods) gives significant *p* values at 40 and 45 s (*p* = 0.025 at 40 s and *p* = 0.030 at 45 s), but does not show significant differences at other timepoints (*p* = 0.055 at 50 s and *p* > 0.15 at all other timepoints); *n* = 6 mice per genotype. ***C***, Novel female interest assay experimental design. A novel female mouse (unfamiliar to the male mice) was randomly placed inside one of the four slotted metallic enclosures placed at the corners of the testing arena. Male mice were placed at the center of the arena facing the visual cues on the wall and subjected to 10 min of experience trial. ***D***, Female interest was scored as the amount of time (out of 10 min total) that the male mouse spent interacting with the female inside the enclosure; *n* = 8 mice per genotype. Error bars represent SEMs.

Visual defects can have a significant impact on the outcomes of behavioral tests. For example, an alternate interpretation to the looming assay result could be that *Tppp* KO mice are not able to visually perceive the looming stimulus. To rule out the possibility of a vision deficit, we used the PLR test to measure the pupil dilation response on the contralateral eye (Fig. 6*A*). Our results show that *Tppp* KO mice display a PLR response with no significant differences compared with their wild-type counterparts (Fig. 6*B*; Extended Data Fig. 6-1). These results show that *Tppp* KO mice dilate pupils following exposure to high amounts of light and suggest that *Tppp* KO mice are not grossly visually impaired.

To further assess whether *Tppp* KO mice have sensory or social deficits, we introduced them to the novel female interest assay (Fig. 6*C*). In this paradigm, mice rely on visual and olfactory cues to detect the presence of the novel female. *Tppp* KO mice displayed no difference from wild-type animals in interaction time (Fig. 6*D*; Extended Data Fig. 6-1). Thus, this indicates that *Tppp* KO mice are neither visually or sensorially impaired in their detection of the novel female nor socially impaired in their interest in the novel female.

10.1523/ENEURO.0170-20.2020.f6-1 Extended Data Figure 6-1 Detailed results of statistical analysis in Figure 6. Download Figure 6-1, XLSX file.

## Discussion

By performing a number of behavioral assays, we show that the hypomyelinated *Tppp* KO mouse displays a specific subset of behavioral deficits. In open-field and light-dark preference assays, *Tppp* KO mice display similar activity levels and time spent in the light and dark compartments compared with wild-type mice, indicating that they do not display anxiety behavior. However, in both traditional fear-conditioning assays and looming fear assays, *Tppp* KO mice show marked defects in both fear memory and innate fear, respectively. In addition, spatial memory tests support a short-term memory deficit in *Tppp* KO mice. These results are consistent with deficits in fear-based responses, but not in anxiety behavior.

Intriguingly, both learning-based fear and innate fear are altered in *Tppp* KO mice. Defects in learning-based fear are consistent with decreased MBP staining in the cortex and hippocampus as well as shorter sheath lengths in the cortex (Fu et al., 2019). However, the looming fear assay is non-memory dependent, and therefore, these deficits may indicate a myelination defect in the underlying innate fear circuitry. Freezing and hiding in response to visual looming threat involve activation of projections from the ventral midline thalamus (vMT) to the basolateral amygdala (BLA; Salay et al., 2018). Two major pathways connect the amygdala to other brain regions: stria terminalis, an efferent pathway to the basal forebrain and hypothalamus, and the amygdalofugal pathway, containing both afferent and efferent connections with the thalamus as well as with basal forebrain and hypothalamus. In contrast to the stria terminalis, the amygdalofugal pathway is a myelinated white matter tract (Mori et al., 2017). Anatomical patterns of myelination in the amygdalofugal pathway, such as average sheath length, frequency and lengths of unmyelinated axon segments, have yet to be elucidated. This type of data could provide mechanistic insights on why hypomyelinated *Tppp* KO animals display innate fear defects.

Indeed, structural deficits in myelination may affect conduction velocity and do so through several mechanisms, including regulation of myelin sheath length, thickness, or nodal length (Arancibia-Cárcamo et al., 2017). *Tppp* KO oligodendrocytes have shorter sheath lengths by about half when compared with wild-type oligodendrocytes both *in vitro* in 3D cultures as well as *in vivo* in the cortex (Fu et al., 2019). Furthermore, oligodendrocytes cultured from different regions, such as cortex and spinal cord, have intrinsic differences in sheath length (Bechler et al., 2015). Thus, brain regions with characteristically long myelin sheaths may be more adversely affected in *Tppp* KO mice. In the amygdalofugal pathway, if myelin sheaths are characteristically long, then shorter sheath lengths in *Tppp* KO mice could contribute to looming fear defects.

Our observations of short-term fear memory deficits in *Tppp* KO mice are consistent with recent reports connecting myelination to long-term fear memory deficits. In wild-type mice, fear learning induces proliferation of OPCs in the medial PFC (mPFC), which then maturate over the time course of approximately four weeks. Mice in which the transcription factor MYRF was inducibly and conditionally knocked out in NG2-positive OPCs display striking defects in freezing in fear conditioning assays on the timescale of 30 d postconditioning, but not at 1 d postconditioning (Pan et al., 2020). This result was independently replicated with similar dramatic defects in freezing when tested 28 d following initial training. Mechanistically, local field potential recordings indicate increases in synchronization in the hippocampal-cortical circuit following fear learning, which is deficient in mutant mice (Steadman et al., 2020). These studies indicate that long-term memory consolidation involves oligodendrogenesis and new myelin sheaths. In contrast, *Tppp* KO mice display short-term fear memory deficits on the faster timescale of 1–2 d, likely consistent with preexisting structural defects in myelination.

Interestingly, other mouse models with structural myelin defects also display behavioral issues. CNP forms the “struts” of cytoplasmic channels that spiral around the myelin sheath (Snaidero et al., 2017) and contain lamellar microtubules (Snaidero et al., 2014). Compared with wild-type mice, heterozygous *Cnp*-null mice spend >3 times the amount of time holding on to a bar, in an assay that models catatonia, a symptom in schizophrenia. However, unlike *Tppp* KO mice (Fu et al., 2019), heterozygous *Cnp*-null mice do not display defects in Rotarod or fear conditioning (Hagemeyer et al., 2012). In addition, behavioral assays performed on mice lacking PLP (proteolipid protein), a transmembrane myelin protein, demonstrated that, in terms of motor coordination, *Plp-*null mice display no Rotarod deficits, but have gait and swimming defects. Behaviorally, *Plp*-null mice spend more time in the open arm of the zero maze and less time burying marbles, but, like *Tppp* KO mice, they displayed no open field assay differences. This specific subset of phenotypes can be attributed to regionally specific enhanced proliferation, as observed in the olfactory bulb and rostral corpus callosum (Gould et al., 2018). Thus, different mouse models of hypomyelination can display distinct subsets of defects; this indicates that future studies should not only perform multiple behavioral assays but should also consider regionally distinct effects in the brain.

Since individual tasks rely on many different brain regions, it is difficult to pinpoint the most affected regions. In *Plp*-null mice, task-specific differences may be attributed to regionally specific increases in oligodendrocyte density in the olfactory bulb and rostral corpus callosum (Gould et al., 2018). In *Tppp* KO mice, hypomyelination has been observed in many regions, including cortex, hippocampus, caudate, and spinal cord (Fu et al., 2019). Thus, TPPP deficiency may broadly affect oligodendrocyte function and contribute to multiple behavioral problems found in *Tppp* KO mice.

The emerging principle of myelin plasticity or adaptive myelination encompasses structural myelin changes following neuronal activity. This has been tested in many paradigms, both after learning and with social environment change. In long-term fear-based learning, oligodendrogenesis and increased myelination play a role in spatial learning and fear-based learning paradigms for periods of up to 30 d (Pan et al., 2020; Steadman et al., 2020; Wang et al., 2020). Mechanistically, learning can strengthen hippocampal-cortical circuits, likely via synchronization of impulse conduction (Freeman et al., 2016; Steadman et al., 2020). For motor learning, both mice subject to optogenetic stimuli and complex wheel activity display changes in myelin structure (Gibson et al., 2014; McKenzie et al., 2014; Xiao et al., 2016). In addition, mice housed in social isolation demonstrate changes in white matter (Liu et al., 2012) and mice subject to chronic social stress have decreased expression of oligodendrocyte genes and myelination (Lehmann et al., 2017; Cathomas et al., 2019). Thus, myelin plasticity may be crucial for behavioral responses to environmental changes and future studies should investigate the molecular involvement of TPPP in microtubule-based myelin structural in response to learning.

## Synthesis

Reviewing Editor: Deanna Smith, University of South Carolina Decisions are customarily a result of the Reviewing Editor and the peer reviewers coming together and discussing their recommendations until a consensus is reached. When revisions are invited, a fact-based synthesis statement explaining their decision and outlining what is needed to prepare a revision will be listed below. The following reviewer(s) agreed to reveal their identity: NONE.

Both reviewers agree that this a well-written manuscript containing new information on behavioral differences between hypomyelinated Tppp knockout mice and controls. The revised manuscript is improved by the inclusion of data to support the specificity of the expression pattern and the statistical analysis.
